# Experiences of obstetric nurses and midwives receiving a perinatal bereavement care training programme: A qualitative study

**DOI:** 10.3389/fmed.2023.1122472

**Published:** 2023-03-15

**Authors:** Jialu Qian, Shuyi Chen, Cecilia Jevitt, Shiwen Sun, Man Wang, Xiaoyan Yu

**Affiliations:** ^1^Faculty of Nursing, Zhejiang University School of Medicine, Hangzhou, China; ^2^Division of Midwifery, Department of Family Medicine, Faculty of Medicine, University of British Columbia, Vancouver, BC, Canada; ^3^Department of Thyroid and Breast Surgery, Huashan Hospital Affiliated to Fudan University, Shanghai, China; ^4^Department of Obstetrics, Women’s Hospital, School of Medicine, Zhejiang University, Hangzhou, China

**Keywords:** bereavement care, qualitative, pregnancy loss, medical education, training

## Abstract

**Aim:**

To explore obstetric nurses and midwifery professionals’ experiences with the Perinatal Bereavement Care Training Programme (PBCTP) after implementation.

**Design:**

A qualitative descriptive design was used.

**Method:**

This qualitative study was conducted at a tertiary level maternity hospital in China. The PBCTP was implemented at Women’s Hospital School of Medicine, Zhejiang University from March to May 2022. A total of 127 nurses and 44 midwives were invited to participate in the training. Obstetric nurses and midwives studied a 5-module training programme comprised of eight online theoretical courses and submitted a reflective journal after each session. Semi-structured interviews were conducted with 12 obstetric nurses and four midwives from May to July 2022 as a post-intervention evaluation. Thematic analysis was used in data analysis.

**Findings:**

A total of 16 participants in this study ranged in age from 23 to 40 years [mean age (SD), 30 (4) years]. Six main themes within participants’ experiences of PBCTP intervention were identified: participants’ aims of undertaking the training; personal growth and practice changes after training; the most valuable training content; suggestions for training improvement; directions for practice improvement; influencing factors of practice optimization.

**Conclusion:**

Nursing and midwifery professionals described the PBCTP as satisfying their learning and skills enhancement needs and supporting positive changes in their care providing for bereaved families. The optimized training programme should be widely applied in the future. More efforts from the hospitals, managers, obstetric nurses, and midwives are needed to jointly contribute to forming a uniform care pathway and promoting a supportive perinatal bereavement care practice.

## 1. Introduction

Perinatal loss occurs worldwide in 25% of known pregnancies and includes miscarriage, stillbirth, therapeutic abortion, and neonatal death (1–3). Women who experience perinatal loss often receive unsatisfactory care, feeling that the care they received did not meet their expectations (4). An online survey done in 44 high-and middle-income countries reported that 25.4% of 3,769 bereaved parents reported disrespectful care and 23.5% reported disrespectful care of their baby (5).

Nurses and midwives play an important role in providing education, assistance, and treatment options to the women who experience perinatal loss (6). When the bereaved families experience congruence between the care they expect and the care they actually receive, the intensity of their grief is lower (7). However, nursing and midwifery professionals reported that they lacked sufficient confidence and capability to provide perinatal bereavement care (8–10), which may have a significant influence on the bereaved families’ mental health (11–13). Nursing and midwifery professionals themselves bear a heavy emotional burden during the provision of perinatal bereavement care (14–16). It may further hinder the quality of care they offer (17, 18). Thus, the gap between women’s needs for high-quality care and nursing and midwifery professionals’ lack of perinatal bereavement care capability has become increasingly prominent (17, 19, 20).

A series of educational programmes in the UK, USA, and Ireland identified in a scoping review sought to prepare nurses and midwives to care for the bereaved parents (21). However, the programmes reviewed lacked a theoretical base and comprehensive training contents. Relevant guidelines have addressed the necessity of conducting continuous training for healthcare professionals in the field of perinatal bereavement care (22–24). Systematic and comprehensive perinatal bereavement care has not been developed in China. Therefore, there was urgency to establish and conduct the Perinatal Bereavement Care Training Programme (PBCTP) to prepare competent nursing and midwifery professionals to provide this specialized care in China. This qualitative study aimed to understand nursing and midwifery professionals’ experiences with the PBCTP after its implementation at a tertiary level maternity hospital in China.

## 2. Materials and methods

### 2.1. Study design

A qualitative descriptive study design was used to record and analyze obstetric nurses’ and midwives’ experiences with the PBCTP. This approach is appropriate for research questions that aim to give a straightforward report of participant experience (25, 26). This study was approved by the Women’s Hospital School of Medicine, Zhejiang University (IRB no. 20210091) Institutional Review Board. Results are reported according to the Consolidated Criteria for Reporting Qualitative Research (COREQ) reporting guideline for qualitative studies (27).

### 2.2. The PBCTP intervention

The PBCTP intervention was specifically designed for nursing and midwifery professionals who provide perinatal bereavement care to parents who experience a perinatal loss. It aimed to meet nursing and midwifery professionals’ knowledge and skills gaps and emotional support needs. There are five modules in this programme including programme introduction, general knowledge of perinatal bereavement care, practical skills of perinatal bereavement care, emotional support for nurses and midwives, and practices reflection and learning. Content was delivered *via* eight online theoretical courses, which were given every 2 days. Asynchronous online lectures, videos, workshops, mindfulness training, and other Supplementary material, were used in the teaching. Follow-up and questions were addressed through the WeChat communications app. More details of the PBCTP Intervention are described in this reference Qian et al. (28) and Supplementary Table 1. Participants received a WeChat prompt after each online course to submit a journal reflection. The aim of the reflective journal was to ensure participants’ completion of the course. Mandarin was the language used in the PBCTP and reflective journal.

### 2.3. Setting and participants

This study was conducted in a delivery room and seven obstetric wards in a tertiary maternity hospital. There are approximately 140 hospital obstetric nurses and 65 midwives working in the study hospital. Nurses and midwives were enrolled in the PBCTP intervention between March and May 2022. The inclusion criteria were nurses or midwives (1) who have provided perinatal bereavement care and (2) who have over 1 year of nursing or midwifery experience. Nurses and midwives who could not complete the training or had received prior perinatal bereavement training were excluded.

### 2.4. Sampling and data collection

**FIGURE 1:**
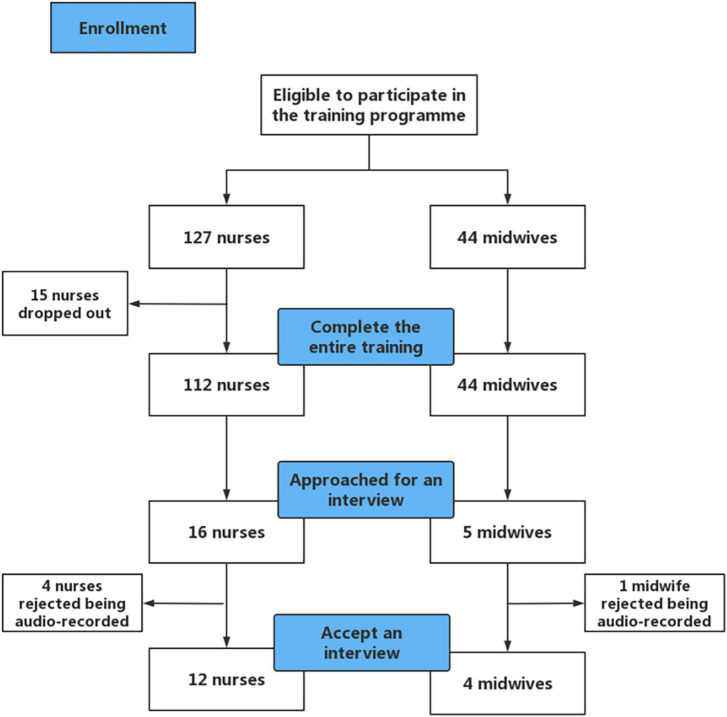
Flow diagram of participants’ inclusion.

Figure 1 shows an overview of participants’ inclusion. Purposive sampling was used to include both nurses and midwives who submitted eight reflective journal entries, and gave detailed, reflective comments in their journals instead of using a few simple words. After the PBCTP intervention, nurses and midwives received a phone call from the researchers (SS) inviting them to participate in the interview. Interviews were conducted *via* telephone using a semistructured interview guide (see Supplementary Table 2) and lasted 30 to 50 min. The interviews were conducted in Mandarin. At the beginning of the interview, the first author (JQ), provided a clear explanation of the objectives of the interview and informed participants about the sound recording. Written and verbal consents were obtained. Member checking was used to ensure the credibility of results (29).

### 2.5. Data analysis

Audio recordings were transcribed verbatim by the first author with personal identifiers removed. *A priori* codes were not used. Themes were identified through a six-stage thematic analysis (data familiarization, initial codes generation, searching for themes, carefully reviewing themes, defining and naming themes, and generating the report) (30). The data analysis was performed from August to October 2022. To fully understand the interviewees’ experiences, the transcripts were read multiple times. Authors (JQ and MW) independently performed line-by-line coding to identify initial concepts adopting inductive and deductive theme approaches. In order to produce the initial codes, each code was highlighted with a different color to identify the most significant and meaningful elements. Then, codes were organized into meaningful categories. Higher-level clusters of categories were generated based on the associations between the codes and categories. Authors (JQ and MW) examined if the themes worked in relation to the coded extracts. After that, clear definitions for each theme were generated. Any disagreement was discussed by all the authors until consensus was reached. Data were managed using NVivo software, version 12 (QSR International).

## 3. Results

### 3.1. Demographic characteristics

We interviewed 12 (75%) hospital obstetric nurses and 4 (25%) midwives. One participant was male. The average age of participants was 30 years. All the participants held a baccalaureate degree. Five of the participants (31%) had ≤5 years clinical experience, 7 (44%) had 6–9 years clinical experience, and 4 (25%) had more than 10 years clinical experience.

Six dominant themes and subthemes were identified within our interviewees’ experiences with the PBCTP intervention, which are shown in Supplementary Table 3 with illustrative quotes.

### 3.2. Theme 1: Participants’ aims of undertaking the training

#### 3.2.1. Needs for knowledge and skill enhancement

Nursing and midwifery staff [6 participants (38%)] stated that in their clinical practice more and more women experience perinatal loss and their needs for knowledge and skill enhancement were proposed. Participants described that they were overwhelmed when encountering such situations. One participant claimed that she lacked communication skills with the bereaved women and their families. Nurses and midwives claimed that the lack of perinatal bereavement care ability made it difficult to obtain trust and cooperation of the women, which hinders their work efficiency.

*“For such patients, we do not have a good way to cope with their emotional changes, which leads to some problems in mutual communication. It is difficult to get their cooperation, which will cause great problems for our work, so I think this course could help us to get a better sense of trust from the women” Nurse 8.*

#### 3.2.2. Providing high-quality care

Due to sympathy for the bereaved women, nursing, and midwifery professionals [7 participants (44%)] instinctively wanted to give assistance to the women as much as possible. They wanted to alleviate women’s negative emotions by providing high-quality care. They wanted to ameliorate women’s negative feelings by providing high quality, emotionally sensitive care. However, the pervasive grief surrounding loss often caused care providers’ usual communications to trigger more unpleasant feelings for women, resulting in dissatisfaction with the nursing and midwifery staff. Nurses and midwives realized that they needed to receive training to offer targeted care for the women and improve their satisfaction. They considered the PBCTP helpful to provide better humanistic care for the women.

*“I found that a large part of the low satisfaction is from women who experienced perinatal loss. This kind of patient is particularly sensitive. If they do not receive proper care, they may have low opinion of the care” Nurse 8.*

### 3.3. Personal growth and practice changes after training

#### 3.3.1. Caring from various perspectives

After training, nursing and midwifery staff [11 participants (69%)] improved their awareness of providing perinatal bereavement care and learned to care women from various perspectives. They began to pay attention to the various needs of the bereaved women, including emotional, spiritual, and other needs. Nurses and midwives more actively asked about women’s needs, paid attention to the expression of language and showed sympathy to the women. They started to provide care based on the actual needs of women instead of their personal views. Nursing and midwifery staff seldom thought of providing care from the perspective of the grieving families’ needs before the PBCTP. Following the PBCTP, they more easily integrated the families’ psychological needs into their care. They thought that they should provide support for the entire family because of the important roles of family members in the women’s psychological recovery.

*“We can provide some help to family members, such as some emotional comfort. Because the support from family members is more important than medical staff. Some family members have a bad mood. It may bring women pressure” Nurse 7.*

#### 3.3.2. Attaching importance to pain management

Some nursing and midwifery staff thought women who experience perinatal loss would have an easier birth than women who experience a full-term pregnancy with a full-grown baby. They underestimated the need for pain management among this population. After learning the course material, nursing and midwifery staff [6 participants (38%)] realized the importance of pain management and they suggested taking actions such as verbal comfort, physical pain management interventions, and analgesics.

*“In the past, when women requested epidural pain relief, we suggested that they wait and told them that epidural use is not cost-effective and may affect progress of labor. Now we try to meet their analgesic needs as much as possible.” Midwife 3 (Women were eligible for epidural pain relief if the gestational age was 28 weeks or greater. All women could receive pain relieving medications if they were unable to cope with the labor pain).*

#### 3.3.3. More effective communication

Being afraid of communicating with the bereaved women was a notable problem in the care provided before the training. One participant described that she did not know how to start communication. After the training, nursing and midwifery staff [4 participants (25%)] gradually overcame their worries and started their communication by giving detailed health education. They would encourage women to recuperate and give instructions on future pregnancy plans.

*“I didn’t dare to communicate with them before, because I thought it would make them feel depressed. I left in a hurry after every ward inspection. Now when I see the bereaved women and their families, I will tell them the following precautions for labor induction. I have received training and learned many courses, so I have a certain knowledge reserve. I have the confidence and ability to communicate with them” Nurse 2.*

### 3.4. Theme 3: The most valuable training content

#### 3.4.1. Respectful grief care

Nursing and midwifery staff [6 participants (38%)] found the content about grief care very practical. Nurses and midwives were more responsive to the women’s needs for grief care. They learned to ask and respect women’s grief care needs (e.g., provision of baby’s footprint, dressing the baby, holding/seeing the baby, etc.). Nurses and midwives paid more attention to words and the expression of language. They said, “baby” rather than “a small corpse.” When the women wanted to see the baby, they would also carefully prepare the baby’s body for viewing.

*“I usually don’t pay attention to how to name the baby, maybe sometimes I just called the baby “a small corpse.” The mother might be very upset to hear that. After we learned the course materials, we would say the baby’s name. I’ll also double-check if the mother wants to see the baby. We’ll clean the baby when the mother needs to see her baby” Midwife 1.*

#### 3.4.2. Increased medical knowledge

Nurses and midwives [5 participants (31%)] believed that learning more about the medical causes of pregnancy loss would help them better answer women’s questions about pregnancy loss. Their basic education did not prepare them for understanding chromosomal abnormalities or fatal formation defects. The bereavement course provided nurses and midwives with more knowledge about the causes of pregnancy loss, and they felt more confident providing care after the course.

*“I think we need to have more theoretical knowledge. For example, women often ask me questions about their babies who have chromosomal abnormalities. I lack theoretical knowledge in this aspect. I want to know what circumstances may lead to fetal abnormality. I want to learn something about doctors’ theoretical knowledge” Midwife 2.*

#### 3.4.3. Mindfulness breathing

After practicing mindfulness breathing in the course, the nursing and midwifery staff [5 participants (31%)] appreciated the effect of mindfulness breathing on relaxation. Mindfulness breathing, as a convenient skill, was recommended to patients by some nursing and midwifery staff. Nurses and midwives also described that they would use mindfulness breathing to release their negative emotions in life.

*“In the provision of bereavement care, we also have negative emotions due to empathy. Providing empathetic bereavement care causes emotional distress in nurses and midwives. But we should manage emotions by ourselves. I think the mindfulness breathing in the course can be helpful in various events in life! Being immersed in sadness is not conducive to our work” Nurse 1.*

### 3.5. Theme 4: Suggestions for training improvement

#### 3.5.1. Strengthening course interactions

Perinatal loss is a solemn topic. Nursing and midwifery staff reported that they do not communicate much about it. Nurses and midwives [7 participants (44%)] thought that online courses lacked communication and interaction between colleagues. They suggested that offline learning would be more intuitive and in-depth. Everyone could share experiences offline. One nurse suggested carrying out in-person courses so they could get immediate feedback on the questions.

*“The courses are recorded, which is one-sided. If these courses can be carried out via several live broadcasts, the effect would be better. For example, if I don’t get any feedback on a certain question, there may be a knowledge point that I don’t understand. It seems that I have learned it, but I don’t know how to use it” Nurse 8.*

#### 3.5.2. Enriching clinical cases

Nursing and midwifery staff [5 participants (31%)] believed that more clinical cases should be added to the training. Theories and clinical cases should be combined because their understanding of perinatal bereavement concepts is theoretical in most cases, and they have doubts about how to apply specific theoretical knowledge to clinical practice.

*“The course content could add more clinical cases. Some concepts are very theoretical and difficult to understand. Our practical experience is not extensive. It is not clear how to specifically apply it to clinical work” Nurse 8.*

#### 3.5.3. Slowing the frequency of courses

Nursing and midwifery staff [3 participants (19%)] reported that the frequency of the course once every 2 days is a little too frequent. Because of their busy clinical work, participants sometimes forgot to study. Therefore, they suggested a slightly longer interval between the two courses.

*“I think the frequency can be slightly slowed down. When we’re learning, we might feel like we’re starting a new lecture right after we’ve learned a lecture. Sometimes it’s tiring to study this course after work. The interval can be slightly longer” Nurse 12.*

### 3.6. Theme 5: Directions for practice improvement

#### 3.6.1. More appropriate content and form of perinatal bereavement care

The nursing and midwifery staff [4 participants (25%)] thought that bereavement care should be provided according to the needs of the bereaved women to be more easily accepted by them. They described that providing baby footprints as mementos is a good memento. In the process of understanding women’s bereavement care needs, it was necessary to ask about their needs in a way that will not cause additional harm. Using a questionnaire that allowed the women to choose their preferred bereavement care was suggested.

*“It would be nice to give the women a questionnaire about what kind of bereavement care they would like us to provide, such as the care of a small body or a farewell ceremony. Because she would feel (the nurse) presumptuous if you asked her directly” Nurse 3.*

#### 3.6.2. Uniform service consensus

After receiving the training, nursing and midwifery staff [5 participants (31%)] believed that uniform and clear guidelines to support women experiencing a perinatal loss should be developed. Uniform guidelines would facilitate the provision of consistent and supportive care. More feedback is needed to guide the application of painless delivery during induction of labor.

*“It would be better to have a uniform nursing process for this population. For example, what care should be provided for the women? Are there any special needs to be satisfied for them? We prefer something routine and then we could offer other assistance according to our clinical experience” Nurse 5.*

### 3.7. Theme 6: Influencing factors of practice optimization

#### 3.7.1. Busy clinical work

Nursing and midwifery staff [4 participants (25%)] felt that the clinical workload was so heavy that they sometimes did not pay attention to their tone during communication and did not have enough time to provide satisfactory bereavement care. It’s not that they did not respect the women. The nurses and midwives were intellectually and emotionally willing to provide high quality bereavement care, but their own exhaustion sometimes weakened their ability.

*“After all, there are a lot of mothers who come to the department to give birth. Sometimes, when we are busy, we may not pay much attention to the bereaved women’s emotions. Maybe at this time, they will feel even worse” Nurse 9.*

#### 3.7.2. Environmental support from the hospital

Nurses and midwives [3 participants (19%)] felt that after training, it would be better if the hospital could provide a private, undisturbed place for perinatal bereavement care. Because postpartum rooms may be shared rooms, there is no private room for the farewell ceremony at the hospital. The cries of other babies can also affect the mood of the bereaved women. In rooms shared by several women, the women often do not want to share their experiences with professionals.

*“If we give a woman who experiences the perinatal loss a separate room, she might be more willing to talk. Another important reason to a provide private room is that the bereaved women don’t want to hear other babies’ voices, because these voices are a stimulus to her” Nurse 3.*

## 4. Discussion

In this qualitative study, nurses and midwives described the PBCTP as supporting growth and changes in their behaviors of providing perinatal bereavement care. Nursing and midwifery staff reported the most valuable training content and suggestions for improving the training. Directions for practice improvement and relevant influencing factors were also proposed.

The reason why nursing and midwifery staff were willing to participate in the PBCTP was that they realized their need for skills enhancement in perinatal bereavement care. This finding is similar to previous research where nurses and midwives also felt inadequate in terms of genetic knowledge, psychological counseling, and communication skills (8, 31). They wished to enhance their ability to provide sufficient support for the bereaved women (9, 32, 33). One survey study found that training in perinatal bereavement care was an important influencing factor in nursing and midwifery staff’s confidence of providing bereavement care (16). Therefore, our study and others highlight the significance of conducting this bereavement training among nurses and midwives.

The findings that nursing and midwifery professionals perceived personal growth and practice changes after training are consistent with other studies implementing this kind of training (34, 35). Positive feedback reflected the effectiveness of the PBCTP. The PBCTP intervention included comprehensive training content, based on a systematic scoping review (21) and the exploration of Chinese perinatal bereavement care (19), which enhanced the evidence-based rigor of the programme. Therefore, wider application and testing of the PBCTP intervention should be conducted with larger samples and in diverse communities to verify its effectiveness.

Respectful grief care increased medical knowledge and mindfulness breathing were considered as the most valuable contents of the training. In China, it is taboo to talk about death. Nurses and midwives were unfamiliar with providing respectful grief care. Nursing and midwifery staff in our study considered asking women’s desire to see the baby after the delivery (36), calling a baby by name, and keeping baby’s footprint (22) in this training as practical bereavement skills. In a previous study, nurses and midwives recommended training related to physical care issues and knowledge of genetics (37), which is similar to our study findings where nursing and midwifery staff acknowledged the value of learning more medical knowledge related to perinatal loss. Richer medical knowledge reserves ensure sufficient confidence to provide perinatal bereavement care.

Moreover, nursing and midwifery staff pointed out the value of practicing mindfulness breathing. A cross-sectional survey of 571 nurses and midwives showed approximately 50% reported average or severe secondary traumatic stress symptoms and emotional exhaustion after responding to providing perinatal bereavement care (16). Mindfulness training has been widely used for relieving healthcare professionals’ traumatic psychological symptoms (38, 39). Mindfulness breathing in our training helped nursing and midwifery professionals to relieve negative emotions.

Due to the COVID-19 pandemic, the PBCTP intervention was implemented *via* online lectures. It reduced the course interactions to some extent. A previous study found that blended learning using a mixture of online resources and materials combined with face-to-face meetings, and in-class interaction had active engagement (40). Organizing offline workshops and debriefing (41, 42) might enhance the learning effects in the future training. Case presentations and case-based discussions could help trainees to strengthen their understanding of abstract concepts (43). As suggested by nurses and midwives in our study, adding more clinical cases in the training could facilitate the combination of theory and clinical practice.

More appropriate content and form of perinatal bereavement care were suggested. Considering the sensitivity of providing perinatal bereavement care, it is important to evaluate women’s bereavement needs in an appropriate way. The key to providing perinatal bereavement support is to respect women’s preferences (44). Hospitals could develop tools such as a list of bereavement care services to help clinical nurses standardize the work process. It could help nursing and midwifery professionals better understand women’s bereavement care needs in a way of avoiding awkwardness (45).

Busy clinical work and environmental support from the hospital were considered factors that limited practice optimization. In this study, nurses and midwives suggested slowing the frequency of courses. That suggestion reflected their busy clinical work. It is similar to previous findings where nurses and midwives also reported the heavy clinical workload was an influencing factor in the bereavement care they provided (19). The improvement of bereavement care involves the contribution of individuals, teams, institutions, and systems (46). Organizational support is necessary to create conditions that enable nursing and midwifery professionals to provide high-quality bereavement care (20). Effective and feasible knowledge should be integrated into the system and continuously monitored and evaluated to maintain the continuous application of knowledge (47). Hospital managers should optimize human resource allocation and create the necessary conditions to enable the implementation, monitoring, and evaluation of best practice perinatal bereavement care (22).

### 4.1. Strengths and limitations

The methodology of this study was rigorous, and a theory-driven approach was adopted for data analysis. This is the first implementation of PBCTP in China and the results were helpful to provide enlightenment about future training for healthcare providers in perinatal bereavement practice.

There are several limitations of this study. The number of participants represented was small. We included nurses and midwives who were expressive in their reflective journals. This purposive sampling may have generated favorably biased interview responses. Additionally, any improvement in perinatal bereavement care is recognized only from the perspectives of the nursing and midwifery professionals, as we did not obtain experiences from the bereaved families after the PBCTP training. Furthermore, the PBCTP was implemented only in a single hospital that included seven wards. Transferability of the findings to other health systems is unknown.

## 5. Conclusion

The PBCTP was described by nursing and midwifery professionals as satisfying their training needs and supporting their professional enhancement. It facilitated personal growth and positive changes in providing perinatal bereavement care. The PBCTP intervention should be optimized based on suggestions from nurses and midwives for wider dissemination and application to verify its effectiveness. More efforts from hospitals, managers, obstetric nurses, and midwives are needed to form a uniform care pathway and promote perinatal bereavement care practice.

## Data availability statement

The original contributions presented in this study are included in the article/Supplementary material, further inquiries can be directed to the corresponding author.

## Ethics statement

The studies involving human participants were reviewed and approved by the Women’s Hospital, School of Medicine, Zhejiang University. The patients/participants provided their written informed consent to participate in this study.

## Author contributions

JQ designed the content of the research, conducted all interviews, carried out the primary analysis, and wrote the manuscript. JQ and MW participated in the data analysis. SS was responsible for participant recruitment and data collection. CJ and XY contributed to the planning of the study and provided feedback on the study design, the results, and drafting of the manuscript. SC was responsible for the revision of the manuscript and consulted in data analysis. All authors were responsible for reviewing the results and contributed to the draft revision.
