# Supplementary material for: Experiences of obstetric nurses and midwives receiving a perinatal bereavement care training programme: A qualitative study
Source: Front Med (Lausanne). 2023 Mar 15;10:1122472. doi: 10.3389/fmed.2023.1122472 (PMC10056219; doi:10.3389/fmed.2023.1122472)
Supplement: Supplementary file 1 [file Table_1.DOCX]

**Supplementary Table 1. The training contents of the PBCTP**

| **Module** | **Main content** | **Specific content** |
| --- | --- | --- |
| 1. Introduction | 1.1 Content, meaning and objectives of the training | - |
|  | 1.2 Duration and form of the training | - |
| 1. General knowledge of perinatal bereavement care | 2.1 The introduction of clinical guidelines and relevant nursing theory | 2.1.1 Clinical Practice Guideline for Care Around Stillbirth and Neonatal Death |
|  |  | 2.1.2 Introduction of Chronic Grief Theory |
|  | 2.2 Knowledge of law and ethics | 2.2.1 Domestic laws, regulations and ethical issues related to perinatal bereavement |
|  | 2.3 Psychological characteristics and needs of bereaved families | 2.3.1 Psychological characteristics of grieving women in different stages of perinatal period |
|  |  | 2.3.2 Various needs of the bereaved women |
|  |  | 2.3.3 Psychological characteristics of bereaved family members (grieving fathers, bereaved sibling, etc.) |
|  |  | 2.3.4 Watching the video of “The Heartbroken Mother: A Letter to My Doctor” |
|  | 2.4 Perinatal bereavement care based on different cultures | 2.4.1 Examples of the special beliefs and practices of death according to different cultural/religious background groups |
| 1. Practical skills of perinatal bereavement care | 3.1 Communication skills and contents | 3.1.1 Basic principles of effective communication |
|  |  | 3.1.2 Role playing of common communication situations in perinatal bereavement care |
|  |  | 3.1.3 Communicating with grieving families：Do say and do not say |
|  | 3.2 Psychological support strategies for the bereaved parents | 3.2.1 Information support throughout the prenatal, delivery and postpartum period |
|  |  | 3.2.2 Enhancing living nursing |
|  |  | 3.2.3 Physical contact and verbal encouragement |
|  |  | 3.2.4 Improving the quality of company |
|  |  | 3.2.5 Strengthening the management of labour progress |
|  | 3.3 Grief care during the pregnancy termination | 3.3.1 Asking and respecting grieving needs of bereaved women (e.g., seeing/hugging the baby, dressing, keeping mementos, etc.) |
|  |  | 3.3.2 Matters needing attention in providing grief care |
|  | 3.4 Skills of labour pain management | 3.4.1 Improving pain management awareness |
|  |  | 3.4.2 Nonpharmacological interventions for labour pain, including supportive interventions, music therapy, acupoint stimulation, etc. |
|  | 3.5 Support strategies for postpartum fatigue | 3.5.1 Postpartum exercise guidance |
| 1. Emotional support for nurses and midwives | 4.1 Introduction of secondary traumatic stress among health care professionals | 4.1.1 Symptoms and self-assessment of secondary trauma |
|  | 4.2 Emotion management and relaxation techniques | 4.2.1 Writing therapy: three good things |
|  |  | 4.2.2 Mindfulness training intervention |
|  | 4.3 Provision of psychological support resources and platforms | 4.3.1 Strengthening peer support among colleagues via group workshops |
|  |  | 4.3.2 Establishing psychological support groups and providing accessible psychological counselling services |
| 1. Practices reflection and learning | 5.1 Group workshop | 5.1.1 Experience sharing and interactive exchange between nursing professionals |
|  | 5.2 Practical reflection conference | 5.2.1 Feedback on clinical practices and issues |
|  |  | 5.2.2 Summarizing the experiences and feelings in the training |
