# Supplementary material for: Experiences of obstetric nurses and midwives receiving a perinatal bereavement care training programme: A qualitative study
Source: Front Med (Lausanne). 2023 Mar 15;10:1122472. doi: 10.3389/fmed.2023.1122472 (PMC10056219; doi:10.3389/fmed.2023.1122472)
Supplement: Supplementary file 2 [file Table_2.DOCX]

**Supplementary Table 2. Phone Interview Guide**

- Is any changes in your provision of care for women who experience perinatal loss after receiving this perinatal bereavement care training programme (PBCTP)?
- What motivates you to participate in PBCTP?
- Have you perceived any improvement after the training?
- Which course is the most helpful or impressive?
- Is there any communication between colleagues in regard with perinatal bereavement care during the training?
- Do you think any other content should be added to the PBCTP?
- Is there any suggestions for improving our training programme?
- Do you think this kind of training should be widely carried out in obstetrics specialized hospitals in the future?
