# Supplementary material for: Experiences of obstetric nurses and midwives receiving a perinatal bereavement care training programme: A qualitative study
Source: Front Med (Lausanne). 2023 Mar 15;10:1122472. doi: 10.3389/fmed.2023.1122472 (PMC10056219; doi:10.3389/fmed.2023.1122472)
Supplement: Supplementary file 3 [file Table_3.DOCX]

**Supplementary Table 3. Themes, Subthemes, and Illustrative Quotes**

| **Themes and subthemes** | **Illustrative quotes** |
| --- | --- |
| Theme 1: Participants’ aims of undertaking the training |  |
| Needs for knowledge and skills enhancement | “I think it is very necessary to carry out this kind of training. I used to be overwhelmed when I met bereaved women. But now I have learned a lot after the training. Every obstetric department may encounter this population, so it is necessary to carry out comprehensive training among nursing staff.” Nurse 2 |
| Providing high-quality care | “It is a pity that some women have to experience induction of labor. I am quite emotional and I want to study further in this field. Then I can provide better care for them in the future.” Nurse 4 |
| Theme 2: Personal growth and practice changes after training |  |
| Caring from various perspectives | “Before, I used to provide care from a clinical perspective. Now, I will consider issues from the women’s point of view. I have more empathy for the women now.” Midwife 4 |
| Attaching importance to pain management | “We didn’t realize the women had such a high need for managing labor pain before, and we didn’t pay attention to it. Nurses and midwives should take measures to deal with women’s pain, such as giving emerol early.” Nurse 3 |
| More effective communication | “Now we'll tell them if there's anything unpleasant, you can tell us. Just like what is said in the course, we can help them to make a new birth plan and make preparations for the future pregnancy.” Nurse 10 |
| Theme 3: The most valuable training content |  |
| Respectful grief care | “After the delivery, we didn’t recommend the mother to see the baby before, and felt that it would do psychological damage to the mother. Now we will satisfy them as much as possible if they want to see the baby. We will inquire about the mother’s grief needs actively ” Midwife 3 |
| Increased medical knowledge | “We didn’t know what went wrong with perinatal loss in particular. There is a course on professional knowledge explaining causes of perinatal loss. It is good to supplement professional medical knowledge.” Nurse 4 |
| Mindfulness breathing | “I feel that the mindfulness breathing is useful, because it is convenient to practice. I teach the women. I also practice it myself. I followed the video and felt relaxed a lot.” Nurse 11 |
| Theme 4: Suggestions for training improvement |  |
| Strengthening course interactions | “In fact, it would be better to organize one or two offline courses. I feel direct communication with colleagues is better. We can talk about different clinical cases of these induced labors, because the situations every nursing staff encounters are not the same” Nurse 7 |
| Enriching clinical cases | “Of course, there are some courses with clinical cases, but some courses do not have cases. If there are sufficient clinical cases, it would be better to understand the training contents and the courses would become more vivid.” Nurse 6 |
| Slowing the frequency of courses | “The (our) clinical work is too busy. Although I study every few days, I still feel that I am studying every day. In addition to the training, we also have the hospital’s vocational study and nursing ward round and other kinds of learning.” Nurse 9 |
| Theme 5: Directions for practice improvement |  |
| More appropriate content and form of perinatal bereavement care | “For newborns, we may print a footprint to commemorate. For women who experienced perinatal loss, we can also provide the footprints for their babies, because this commemoration is what we could do for the mothers. It won’t hurt them and they could accept it. In regard to seeing the babies after delivery, some women may refuse.” Nurse 9 |
| Uniform service consensus | “After this training, it is better to clarify what improvement measures have been implemented in the hospital, such as what service you can provide in the delivery room and what specific improvements have been made. So that it can be actually applied to our work, and we can really improve the service for these women.” Nurse 3 |
| Theme 6: Influencing factors of practice optimisation |  |
| Busy clinical work | “Manpower is limited, which can’t be helped. The workload is also very heavy. In fact, many clinical issues cannot be completely satisfactory.” Nurse 4 |
| Environmental support from the hospital | “If there is a quiet environment to say goodbye to the baby, that is good. We hope that we can improve the physical environment for the women to enhance their experiences of clinical care.” Nurse 3 |
